# Sleep Characteristics and Long-Term Risk of Type 2 Diabetes Among Women With Gestational Diabetes

**DOI:** 10.1001/jamanetworkopen.2025.0142

**Published:** 2025-03-05

**Authors:** Xin Yin, Wei Bao, Sylvia H. Ley, Jiaxi Yang, Sherri Babaeian Cuffe, Guoqi Yu, Jorge E. Chavarro, Peipei Liu, Juan Helen Zhou, Deirdre K. Tobias, Frank B. Hu, Cuilin Zhang

**Affiliations:** 1 Global Centre for Asian Women’s Health, Yong Loo Lin School of Medicine, National University of Singapore, Singapore; 2 Department of Obstetrics and Gynaecology, Yong Loo Lin School of Medicine, National University of Singapore, Singapore; 3 Bia-Echo Asia Centre for Reproductive Longevity and Equality, Yong Loo Lin School of Medicine, National University of Singapore, Singapore; 4 Division of Life Sciences and Medicine, University of Science and Technology of China, Hefei, Anhui, China; 5 Department of Epidemiology, School of Public Health and Tropical Medicine, Tulane University, New Orleans, Louisiana; 6 Department of Nutrition, Harvard T.H. Chan School of Public Health, Boston, Massachusetts; 7 Department of Epidemiology, Harvard T.H. Chan School of Public Health, Boston, Massachusetts; 8 Channing Division of Network Medicine, Department of Medicine, Brigham and Women’s Hospital and Harvard Medical School, Boston, Massachusetts; 9 Centre for Sleep and Cognition, Yong Loo Lin School of Medicine, National University of Singapore, Singapore; 10 Department of Medicine & Human Potential Translational Research Program, Yong Loo Lin School of Medicine, National University of Singapore, Singapore; 11 Division of Preventive Medicine, Brigham and Women’s Hospital and Harvard Medical School, Boston, Massachusetts; 12 Centre for Translational Magnetic Resonance Research, Yong Loo Lin School of Medicine, National University of Singapore, Singapore

## Abstract

**Importance:**

Women with a history of gestational diabetes (GD) are at high risk for developing type 2 diabetes (T2D). Sleep is a crucial lifestyle factor associated with cardiometabolic health, yet studies on its role in the progression from GD to T2D are sparse.

**Objective:**

To investigate the associations of sleep duration and quality with T2D risk and levels of glucose metabolism biomarkers in women with a history of GD.

**Design, Setting, and Participants:**

This cohort study used data from the Nurses’ Health Study II, an ongoing longitudinal cohort that began in 1989 and initially included 116 429 female nurses with health status and lifestyle factors updated every 2 to 4 years. A subset of participants with a history of GD was followed up through June 2021. Sleep characteristics were assessed in the 2001 questionnaire (administered from June 2001 to June 2003), which served as the baseline for follow-up. Data were analyzed from November 2023 to August 2024.

**Exposures:**

Snoring frequency, sleep duration, and daytime sleepiness.

**Main Outcomes and Measures:**

Physician-diagnosed incident T2D was ascertained biennially via questionnaires. Cox proportional hazards regression models were used to estimate the hazard ratios (HRs) and 95% CIs. Biomarkers (glycated hemoglobin [HbA_1c_], C-peptide, and insulin) were compared across sleep characteristic categories using multivariable-adjusted least-squares means (LSMs) and 95% CIs.

**Results:**

During a total of 42 155 person-years of follow-up among 2891 women with a history of GD (mean [SD] age, 45.3 [4.4] years), 563 women (19.5%) developed T2D. Compared with women who reported rarely snoring, those with occasional or regular snoring had significantly higher T2D risk, with adjusted HRs of 1.54 (95% CI, 1.18-2.02) and 1.61 (95% CI, 1.21-2.13), respectively. Compared with women who slept 7 to 8 hours per day, shorter sleep duration (≤6 hours per day) was significantly associated with a higher risk of T2D (HR, 1.32; 95% CI, 1.06-1.64). Women who slept 6 or fewer hours per day and snored regularly had the highest risk of developing T2D (HR, 2.06; 95% CI, 1.38-3.07). Additionally, more frequent snoring was associated with higher HbA_1c_ (LSM, 5.89; 95% CI, 5.75-6.02), C-peptide (LSM, 4.30; 95% CI, 3.70-4.99), and insulin (LSM, 11.25; 95% CI, 8.75-14.40) levels in the full adjusted models (*P* = .01 for trend for all).

**Conclusions and Relevance:**

In this cohort study of women with a history of GD, shorter sleep duration and both occasional and regular snoring were significantly associated with an increased risk of T2D. These findings suggest that improving sleep health may be important to reduce T2D incidence in this high-risk population.

## Key Points

**Question:**

Are sleep duration and snoring frequency associated with progression of gestational diabetes (GD) to type 2 diabetes (T2D) in women?

**Findings:**

In this cohort study of 2891 women with a history of GD with 42 155 person-years of follow-up, shorter sleep duration and both regular and occasional snoring were significantly associated with an increased risk of T2D. Regular snoring was associated with unfavorable glucose metabolism profiles, including higher glycated hemoglobin, C-peptide, and insulin levels.

**Meaning:**

These findings suggest that improving sleep health is important to prevent progression from GD to T2D in women.

## Teaser

This cohort study assesses whether sleep duration and quality are associated with incidence of type 2 diabetes among women with a history of gestational diabetes who participated in the Nurses’ Health Study II.

## Introduction

Sleep health has emerged as a critical public health issue globally.^1^ In 2020, the Centers for Disease Control and Prevention reported that one-third of US adults regularly slept less than the recommended 7 hours per day.^2^ Over the past decades, there has been a surge in research regarding sleep health and its implications for various health outcomes, including cardiovascular health, cancer, infectious disease, dementia, and depression.^3,4^ Accumulating evidence suggests a positive correlation between frequent snoring and shortened sleep duration and an increased risk of type 2 diabetes (T2D) in the general population.^5,6^ Additionally, poor sleep quality and circadian disruption are associated with increased postprandial plasma glucose levels, increased levels of glycated hemoglobin (HbA_1c_), and insulin resistance.^7,8^

Gestational diabetes (GD) is one of the most common pregnancy complications, affecting approximately 2% to 30% of pregnancies worldwide and 6.5% to 11.9% in the US.^9^ Women diagnosed with GD are nearly 10 times more likely to develop T2D later in life than those without a GD history.^10^ Therefore, it is imperative to identify modifiable lifestyle factors related to the progression from GD to T2D. Women with a history of GD experience β-cell dysfunction and/or insulin resistance during pregnancy that may continue to deteriorate over their lifespan.^11,12^ Due to these underlying biological mechanisms, women with GD often develop T2D several years earlier than the general population.^13^ Consequently, the associations between modifiable lifestyle factors and the risk of T2D in the general population may differ from those observed in this high-risk population, necessitating further research into which lifestyle factors are significant and to what extent. Some previous studies have assessed various modifiable risk factors, including diet quality and physical activity, in relation to the risk of T2D in this high-risk group.^14,15^ However, the association between sleep characteristics and the long-term risk of T2D and glucose metabolic health in these high-risk women remains unclear.

To address this research gap, our study aimed to examine the associations of sleep duration, snoring frequency, and daytime sleepiness with the risk of T2D in women with a history of GD. Furthermore, we investigated whether these sleep-related factors are associated with biomarkers of glucose metabolism, including HbA_1c_, insulin, and C-peptide, in this high-risk population.

## Methods

### Study Population

The population of this cohort study was composed of women with a history of GD in the Nurses’ Health Study II (NHSII), which is part of the Diabetes & Women’s Health (DWH) Study.^16,17^ The DWH Study aims to identify determinants for the progression from GD to T2D. Established in 1989, the NHSII is an ongoing prospective cohort study that initially recruited 116 429 registered female nurses aged 24 to 44 years.^18^ The cohort participates in biennial dissemination of self-reported questionnaires to gather updates on health-related behaviors and disease outcomes, with health status and lifestyle factors updated every 2 to 4 years. The study protocol was approved by the institutional review boards of the Brigham and Women’s Hospital and the Harvard T.H. Chan School of Public Health, with participants’ consent implied by the return of the questionnaires. We followed the Strengthening the Reporting of Observational Studies in Epidemiology (STROBE) reporting guideline.

In this study, we included women who reported a history of GD and answered questions on sleep characteristics in the NHSII 2001 questionnaire (administered from June 2001 to June 2003), which served as the baseline for follow-up. Cases of GD were identified from 1989 to 2001 in the NHSII cohort. The 2001 questionnaire was the last to include questions regarding GD, as most NHSII participants had passed reproductive age. At baseline, we excluded women from the analysis if they (1) had a history of type 1 diabetes, multiple-gestation pregnancies (twins or multiple births), or missing birth dates; (2) had a history of T2D, cardiovascular disease (myocardial infarction or stroke), or cancer before 2001 when sleep characteristics were reported; (3) were diagnosed with T2D prior to GD; (4) had missing data on sleep-related factors; or (5) did not report having a history of GD. These women were followed up biennially until June 2021.

Race and ethnicity, ascertained by self-report, were included in the analysis to examine their association with sleep and the risk of T2D. Because most participants were White, race and ethnicity were categorized as White and other (included American Indian or Native American, Asian, Black, Hawaiian, and multiracial).

### Assessment of Sleep Variables

Sleep characteristics were self-reported only once, in the 2001 baseline questionnaire (details are included in the eMethods in Supplement 1). The participants were asked how many hours, on average, they slept over a 24-hour period and whether they snored. Daytime sleepiness was assessed with the question, “On average, how often are your daily activities affected because you are sleepy during the day?” We recategorized snoring frequency into 3 groups: almost never (0 nights per week), occasionally (1-2 nights per week), and regularly (≥3 nights per week). For sleep duration, we grouped participants into 6 or fewer, 7 to 8, and 9 or more hours per day for the main analysis. Daytime sleepiness was recategorized into rarely or never (0 days per week), 1 to 3 days per week, and at least 4 days per week.

### Ascertainment of T2D

Participants reporting physician-diagnosed T2D on each biennial questionnaire were mailed a supplementary questionnaire regarding symptoms, diagnostic tests, and hypoglycemic therapy to confirm self-reported diagnoses. Confirmed diabetes required meeting at least 1 of the criteria on the supplementary questionnaire as defined by the American Diabetes Association (ADA)^19^ (details are included in the eMethods in Supplement 1).

### Assessment of Biomarkers

Details of the assessment of biomarkers are included in the eMethods in Supplement 1. From 2012 to 2014, 3667 women with a history of GD from the NHSII cohort were invited to the DWH Study for the questionnaire and biospecimen collection.^16,17^ In this study, we assessed HbA_1c_, insulin, and C-peptide levels. In examining the associations between sleep characteristics and glycemic metabolism biomarkers, we excluded women who (1) received a T2D diagnosis prior to the time of blood sample collection, (2) had an HbA_1c_ level of 6.5% or higher at blood sample collection (as suspected undiagnosed diabetes), and/or (3) lacked all glucose metabolism biomarkers of interest (ie, HbA_1c_, C-peptide, and insulin).

### Statistical Analysis

Data were analyzed from November 2023 to August 2024. Details of the covariates assessment and statistical analysis are included in the eMethods in Supplement 1. Follow-up time was calculated from the 2001 baseline to T2D diagnosis, death, the last biennial questionnaire response, or June 2021, whichever came first. We used Cox proportional hazards regression models to estimate hazard ratios (HRs) and 95% CIs for the association between baseline sleep characteristics and the risk of T2D. All Cox proportional hazards regression models were stratified by age (in months) and calendar time and adjusted for parity (1, 2, or ≥3), race and ethnicity (White, other), family history of diabetes (yes, no), oral contraceptive use (never, past, or current), menopausal status (premenopausal, postmenopausal), ever night shift work (yes, no), respiratory illnesses (yes, no), depression (yes, no), antidepressant use (yes, no), use of other medications known to affect sleep (including β-blockers, oral steroids, and minor tranquilizers [yes, no]), cigarette smoking (current, former, or never), physical activity (metabolic equivalent hours per week [quartiles]), total energy intake (quartiles), alcohol intake (quartiles), caffeine consumption (quartiles), Alternate Healthy Eating Index score (quartiles),^20^ and body mass index (BMI) (<21, 21 to <23, 23 to <25, 25 to <27, 27 to <30, 30 to <33, 33 to <35, 35 to <40, or ≥40 [calculated as weight in kilograms divided by height in meters squared]). We conducted a likelihood ratio test comparing models with and without the interaction term between exposure and age as a proxy for calendar time (<45 years or ≥45 years [ie, median]). The nonsignificant likelihood ratio tests indicated no violation of the proportional hazards assumption.

We investigated potential nonlinear associations between sleep duration and the risk of T2D incidence using restricted cubic splines.^21^ Joint associations between sleep characteristics were also tested. We tested each biomarker for normality and applied log transformation where distributions were skewed. Generalized linear models were used to estimate the least-squares means (LSMs) of metabolic biomarker levels across categories of sleep characteristics. To test for linear trends across these categories, we assigned the median value to each category and incorporated this continuous variable into the models. All statistical analyses were performed using SAS, version 9.3 (SAS Institute Inc). Two-sided *P* < .05 was considered statistically significant.

## Results

Among 2891 women with a history of GD and available data on sleep characteristics (mean [SD] age, 45.3 [4.4] years), we documented 563 incident T2D cases (19.5% of participants) over 42 155 person-years of follow-up (mean [SD] follow-up duration, 17.3 [5.1] years). A total of 2681 women (92.7%) were White, and 210 (7.3%) were other race or ethnicity. At baseline (2001), compared with women who rarely or never snored, those who snored regularly were more likely to be current smokers, have respiratory illnesses and depression, and use medications known to affect sleep (Table 1). Regular snorers generally had less physical activity, lower diet quality, higher total calorie intake, and a higher BMI. Moreover, compared with women who slept 7 to 8 hours per day, those with shorter sleep durations (≤6 hours per day) were more likely to be postmenopausal, night shift workers, and have higher BMI, caffeine intake, and prevalence of depression. Compared with women who rarely or never experienced daytime sleepiness, those with a higher frequency of daytime sleepiness were often night shift workers, had a higher likelihood of respiratory illnesses and depression, were more likely to use medications known to affect sleep, and had increased caffeine intake, reduced physical activity, and a higher BMI.

**Table 1.** Age-Standardized Baseline Characteristics of Women With a History of GD According to Sleep Characteristics in the Nurses’ Health Study II

Characteristic	Participants, % (N = 2891)^a^								
	Frequency of snoring			Sleep duration, h/d			Frequency of daytime sleepiness		
	Almost never (n = 1047)	Occasionally (n = 1039)	Regularly (n = 805)	≤6 (n = 910)	7-8 (n = 1830)	≥9 (n = 151)	Rarely or never (n = 1724)	1-3 d/wk (n = 718)	≥4 d/wk (n = 449)
Age, mean (SD), y	44.7 (4.4)	45.7 (4.5)	45.8 (4.5)	45.6 (4.4)	45.2 (4.5)	45.1 (4.3)	45.5 (4.5)	44.9 (4.3)	45.4 (4.4)
Age at first report of GD, mean (SD), y	32.3 (4.9)	32.9 (4.9)	32.8 (5.0)	32.7 (5.1)	32.7 (4.8)	32.0 (4.5)	32.6 (4.9)	32.7 (5.0)	32.7 (5.0)
Race									
White	94.5	91.4	91.3	89.3	94.4	95.0	93.3	92.7	92.0
Other^b^	5.5	8.6	8.7	10.7	5.6	5.0	6.7	7.3	8.0
Family history of diabetes	40.6	44.8	45.2	46.6	40.9	49.2	42.7	41.9	40.9
Parity, median (IQR)	2 (2-3)	2 (2-3)	2 (2-3)	2 (2-3)	2 (2-3)	2 (2-3)	2 (2-3)	2 (2-3)	2 (2-3)
BMI, mean (SD)	25.5 (5.0)	28.2 (5.9)	31.3 (7.1)	28.5 (6.5)	27.6 (6.2)	27.8 (4.6)	27.5 (6.2)	28.0 (6.1)	29.9 (6.7)
Smoking status									
Never	69.9	69.2	62.7	64.8	67.9	68.0	67.2	65.0	69.4
Past	25.4	21.6	23.6	23.6	24.5	23.7	24.5	27.0	21.5
Current	4.8	9.2	13.6	11.6	7.5	8.3	8.4	8.0	9.1
Postmenopausal	15.1	14.4	15.4	17.7	14.7	13.9	15.5	12.4	16.7
Ever night shift worker	7.6	7.8	10.1	9.6	7.3	10.1	7.3	8.8	11.1
Oral contraceptives use									
Never	12.8	11.8	13.3	13.8	12.4	13.1	13.0	12.6	9.9
Past	78.2	79.3	78.3	78.2	78.2	74.6	78.6	77.2	80.3
Current	8.9	9.0	8.5	8.0	9.4	12.2	8.4	10.2	9.8
Respiratory illnesses^c^	12.9	17.6	20.6	18.3	15.3	22.6	13.9	16.8	24.1
Depression	11.8	12.5	18.1	16.7	11.1	20.9	9.2	15.1	26.6
Current use of antidepressants	13.4	19.1	28.1	18.1	18.5	36.7	14.3	20.7	33.8
Current use of other medications known to affect sleep									
β-Blockers	3.4	5.5	7.5	5.9	5.2	8.1	4.8	5.4	6.7
Oral steroids	1.4	2.0	3.0	1.7	1.9	3.3	1.1	2.3	3.7
Minor tranquilizers	3.7	3.5	4.0	5.0	2.9	5.6	2.8	3.6	6.6
Alcohol intake, mean (SD), g/d	3.4 (6.3)	2.8 (5.1)	2.7 (5.3)	2.9 (6.0)	3.2 (5.7)	2.3 (3.6)	3.2 (5.9)	2.9 (4.9)	2.9 (5.9)
Caffeine intake, mean (SD), mg/d	201 (197)	220 (196)	221 (198)	239 (222)	209 (191)	204 (141)	218 (196)	201 (188)	222 (202)
Physical activity, mean (SD), MET-h/wk^d^	24.6 (56.6)	16.8 (23.6)	15.3 (25.9)	24.4 (65.4)	17.7 (24.9)	20.5 (21.2)	20.9 (42.9)	19.3 (31.5)	15.2 (20.7)
Total calories, mean (SD), kcal/d	1834 (540)	1913 (590)	1983 (602)	1890 (586)	1912 (574)	1964 (495)	1856 (573)	2001 (568)	1950 (582)
AHEI score, mean (SD)	46.9 (11.1)	45.3 (10.7)	43.6 (10.2)	45.1 (11.0)	45.7 (10.9)	45.3 (7.7)	45.9 (10.8)	45.3 (10.7)	44.2 (10.9)

Abbreviations: AHEI, Alternate Healthy Eating Index with alcohol component excluded; BMI, body mass index (calculated as weight in kilograms divided by height in meters squared); GD, gestational diabetes; MET, metabolic equivalent.

^a^ Except for age at baseline and age at first report of GD, values were standardized to the age distribution of the study population. Data are presented as age-standardized percentages of participants unless otherwise indicated.

^b^ Included American Indian or Native American, Asian, Black, Hawaiian, and multiracial.

^c^ Included asthma, emphysema, chronic bronchitis, and pneumonia.

^d^ MET values were calculated from the sum of weekly leisure time physical activities of moderate or vigorous intensity; 7.5 MET-hours per week is equivalent to 150 minutes per week of moderate-intensity or 75 minutes per week of vigorous-intensity physical activity.

Frequent snoring was significantly associated with an increased risk of T2D (Table 2). After adjusting for age, parity, family history of diabetes, diet, and other factors, snoring was significantly associated with higher T2D risk, with an HR of 2.17 (95% CI, 1.67-2.80) for occasional snoring and 2.66 (95% CI, 2.04-3.47) for regular snoring compared with almost never snoring (*P* < .001 for trend). The associations remained significant after further adjusting for baseline BMI, with HRs of 1.54 (95% CI, 1.18-2.02) for occasional snoring and 1.61 (95% CI, 1.21-2.13) for regular snoring (*P* = .01 for trend for both).

**Table 2.** Association of Sleep Characteristics With Risk of T2D Among Women With a History of Gestational Diabetes in the Nurses’ Health Study II

Characteristic	HR (95% CI)^a^			*P* value for trend
	**Frequency of snoring**			
	**Almost never**	**Occasionally**	**Regularly**	
T2D cases, No.	120	216	227	NA
Person-years	16 473.6	14 969.6	10 712.3	NA
Cases per 1000 person-years	7.3	14.4	21.2	NA
Age-adjusted model	1 [Reference]	2.13 (1.68-2.71)	2.84 (2.23-3.61)	<.001
Multivariable model	1 [Reference]	2.17 (1.67-2.80)	2.66 (2.04-3.47)	<.001
Multivariable model plus BMI^b^	1 [Reference]	1.54 (1.18-2.02)	1.61 (1.21-2.13)	.01
	**Sleep duration, h/d** ^c^			
	**≤6**	**7-8**	**≥9**	
T2D cases, No.	200	329	34	NA
Person-years	12 428.3	27 495.4	2231.8	NA
Cases per 1000 person-years	16.1	12.0	15.2	NA
Age-adjusted model	1.36 (1.12-1.66)	1 [Reference]	1.15 (0.78-1.71)	NA
Multivariable model	1.33 (1.08-1.65)	1 [Reference]	0.97 (0.63-1.49)	NA
Multivariable model plus BMI^b^	1.32 (1.06-1.64)	1 [Reference]	1.03 (0.66-1.61)	NA
	**Frequency of daytime sleepiness**			
	**Rarely or never**	**1-3 d/wk**	**≥4 d/wk**	
T2D cases, No.	308	143	112	NA
Person-years	25 750.1	10 295.6	6109.8	NA
Cases per 1000 person-years	12.0	13.9	18.3	NA
Age-adjusted model	1 [Reference]	1.06 (0.85-1.32)	1.50 (1.18-1.91)	.002
Multivariable model	1 [Reference]	1.08 (0.85-1.36)	1.29 (0.98-1.70)	.07
Multivariable model plus BMI^b^	1 [Reference]	1.02 (0.80-1.30)	1.06 (0.80-1.41)	.69

Abbreviations: BMI, body mass index (calculated as weight in kilograms divided by height in meters squared); HR, hazard ratio; NA, not applicable; T2D, type 2 diabetes.

^a^ Cox proportional hazards regression models were stratified by age (months) and calendar time and adjusted for parity, race and ethnicity, family history of diabetes, oral contraceptive use, menopausal status, ever night shift work, respiratory illnesses, depression, antidepressant use, use of other medications known to affect sleep, cigarette smoking, physical activity, total energy intake, alcohol intake, caffeine consumption, and Alternate Healthy Eating Index score.

^b^ BMI was modeled as less than 21, 21 to less than 23, 23 to less than 25, 25 to less than 27, 27 to less than 30, 30 to less than 33, 33 to less than 35, 35 to less than 40, and 40 or greater.

^c^ *P* for trend was not tested due to the nonlinear association observed between sleep duration and the risk of T2D; *P* = .01 for nonlinearity.

**Figure 1.**
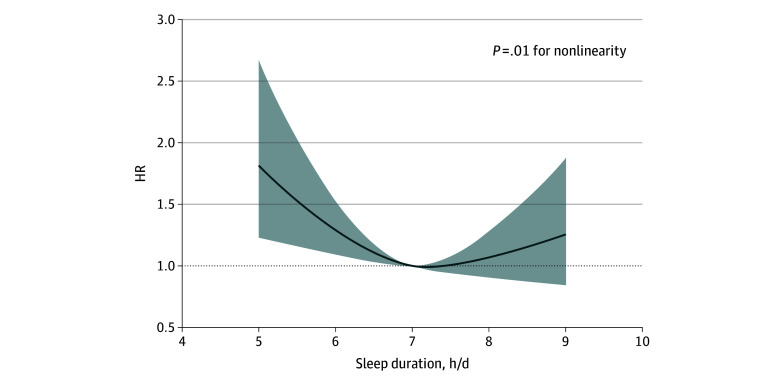
Nonlinear Association of Sleep Duration With Risk of Type 2 Diabetes Among Women With a History of Gestational Diabetes in the Nurses’ Health Study II Cox proportional hazards regression models were stratified by age (months) and calendar time and adjusted for parity, race and ethnicity, family history of diabetes, oral contraceptive use, menopausal status, ever night shift work, respiratory illnesses, depression, antidepressant use, use of other medications known to affect sleep, cigarette smoking, physical activity, total energy intake, alcohol intake, caffeine consumption, Alternate Healthy Eating Index score, and body mass index. Solid line represents the hazard ratio (HR), and shading represents the 95% CI.

Compared with individuals sleeping 7 to 8 hours per day, those who slept less (≤6 hours per day) had a significantly higher risk of T2D after adjusting for all covariates, with an HR of 1.32 (95% CI, 1.06-1.64) (Table 2). In contrast, compared with sleeping 7 to 8 hours per day, long sleep duration (≥9 hours per day) did not show a significant association with T2D risk. In addition, restricted cubic spline analysis suggested a nonlinear U-shaped association between sleep duration and T2D risk (*P* = .01 for nonlinearity) (Figure 1). Compared with sleeping 7 hours per day (reference), shorter sleep duration was significantly associated with an increased risk of T2D.

Women who experienced daytime sleepiness 4 or more days per week had a higher risk of developing T2D (HR, 1.50; 95% CI, 1.18-1.91) in the age-adjusted model compared with those who never or rarely experienced it. However, there was no association after additional adjustments for other covariates (Table 2). Stratified analyses by other factors did not reveal any significant differences in the association between sleep characteristics and the risk of T2D (eTable 1 in Supplement 1).

**Figure 2.**
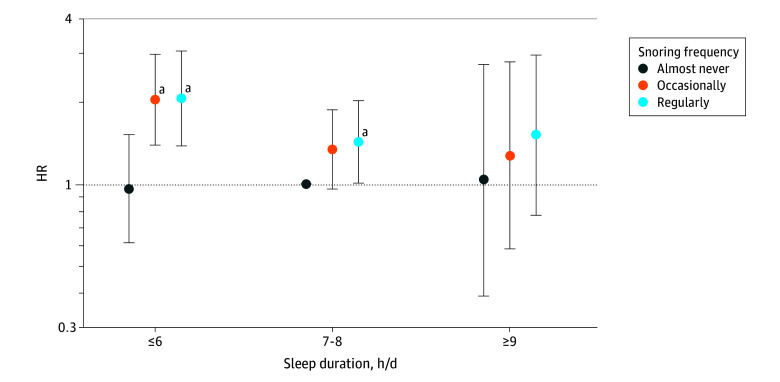
Joint Association Between Sleep Duration and Snoring Frequency and Risk of Type 2 Diabetes Among Women With a History of Gestational Diabetes in the Nurses’ Health Study II Cox proportional hazards regression models were stratified by age (months) and calendar time and adjusted for parity, race and ethnicity, family history of diabetes, oral contraceptive use, menopausal status, ever night shift work, respiratory illnesses, depression, antidepressant use, use of other medications known to affect sleep, cigarette smoking, physical activity, total energy intake, alcohol intake, caffeine consumption, Alternate Healthy Eating Index score, and body mass index. Error bars represent 95% CIs. HR indicates hazard ratio. ^a^*P* < .05.

Women who both slept less and snored regularly had higher risk of T2D (Figure 2 and eTable 2 in Supplement 1). Specifically, compared with women who slept 7 to 8 hours per day and almost never snored, those who slept 6 or fewer hours per day and regularly snored had the highest risk of developing T2D, with an HR of 2.06 (95% CI, 1.38-3.07), followed by those who slept 6 or fewer hours per day and occasionally snored, with an HR of 2.04 (95% CI, 1.39-2.99). Other joint associations are presented in eTables 3 and 4 in Supplement 1.

A total of 527 women with a history of GD who provided both fasting blood samples and sleep characteristics were included in our final biomarker analysis. Women who snored regularly had significantly higher HbA_1c_ levels compared with those who rarely snored (adjusted LSM, 5.89 [95% CI, 5.75-6.02] vs 5.78 [95% CI, 5.64-5.93]; to convert to proportion of total hemoglobin, multiply by 0.01) (Table 3). Higher frequency of snoring was significantly and positively associated with HbA_1c_ levels in all models, including the full adjusted model (LSM, 5.89; 95% CI, 5.75-6.02; *P* = .01 for trend). Snoring frequency was also positively associated with C-peptide (LSM, 4.30; 95% CI, 3.70-4.99) and insulin (LSM, 11.25; 95% CI, 8.75-14.40) levels in the full adjusted model (*P* = .01 for trend for both). No significant association was found between sleep duration and metabolic biomarker levels or between daytime sleepiness and metabolic biomarker levels after adjusting for all covariates.

**Table 3.** Least-Squares Means of Metabolic Biomarker Levels According to Sleep Characteristics Among 527 Women With a History of GD in the Nurses’ Health Study II

Biomarker	Least-squares mean (95% CI)^a^			*P* value for trend
	**Frequency of snoring**			
	**Almost never**	**Occasionally**	**Regularly**	
HbA_1c_, % (n = 523)				
Age-adjusted model	5.57 (5.52-5.61)	5.67 (5.62-5.72)	5.75 (5.69-5.81)	<.001
Multivariable model^b^	5.76 (5.62-5.90)	5.84 (5.70-5.98)	5.92 (5.79-6.06)	<.001
Multivariable model plus BMI^c^	5.78 (5.64-5.93)	5.84 (5.70-5.98)	5.89 (5.75-6.02)	.01
C-peptide, ng/mL (n = 449)				
Age-adjusted model	3.24 (3.08-3.41)	3.74 (3.53-3.96)	4.21 (3.95-4.50)	<.001
Multivariable model^b^	3.63 (3.08-4.27)	4.07 (3.47-4.76)	4.60 (3.94-5.37)	<.001
Multivariable model plus BMI^c^	3.81 (3.26-4.45)	3.99 (3.43-4.64)	4.30 (3.70-4.99)	.01
Insulin, μIU/mL (n = 463)				
Age-adjusted model	6.77 (6.21-7.39)	8.97 (8.15-9.89)	10.84 (9.70-12.10)	<.001
Multivariable model^b^	8.41 (6.41-11.04)	10.70 (8.22-13.92)	13.09 (10.09-16.99)	<.001
Multivariable model plus BMI^c^	9.16 (7.10-11.82)	10.19 (7.95-13.06)	11.25 (8.75-14.40)	.01
	**Sleep duration, h/d**			
	**≤6**	**7-8**	**≥9**	
HbA_1c_, % (n = 523)				
Age-adjusted model	5.66 (5.60-5.72)	5.64 (5.60-5.67)	5.70 (5.57-5.83)	.93
Multivariable model^b^	5.88 (5.74-6.02)	5.87 (5.74-6.01)	5.87 (5.68-6.07)	.96
Multivariable model plus BMI^c^	5.85 (5.72-5.99)	5.85 (5.72-5.99)	5.86 (5.66-6.05)	.87
C-peptide, ng/mL (n = 449)				
Age-adjusted model	3.81 (3.56-4.08)	3.55 (3.41-3.70)	3.91 (3.36-4.56)	.34
Multivariable model^b^	4.38 (3.73-5.15)	4.21 (3.59-4.94)	4.53 (3.59-5.73)	.81
Multivariable model plus BMI^c^	4.15 (3.57-4.83)	4.06 (3.50-4.70)	4.45 (3.58-5.53)	.86
Insulin, μIU/mL (n = 463)				
Age-adjusted model	9.11 (8.12-10.24)	8.01 (7.47-8.58)	10.41 (8.06-13.45)	.52
Multivariable model^b^	12.02 (9.16-15.84)	11.13 (8.51-14.54)	13.81 (9.36-20.45)	.91
Multivariable model plus BMI^c^	10.64 (8.31-13.65)	10.25 (8.03-13.07)	13.02 (9.13-18.57)	.44

Abbreviations: BMI, body mass index (calculated as weight in kilograms divided by height in meters squared); GD, gestational diabetes; HbA1_1c_, glycated hemoglobin.

SI conversion factors: To convert HbA_1c_ percentage of total hemoglobin to proportion of total hemoglobin, multiply by 0.01; C-peptide to nmol/L, multiply by 0.331; and insulin to pmol/L, multiply by 6.945.

^a^ Analyses were conducted among women with a history of GD who did not have type 2 diabetes at the time of blood sample collection.

^b^ Covariates included age (months), parity, race and ethnicity, family history of diabetes, oral contraceptive use, menopausal status, ever night shift work, respiratory illnesses, depression, antidepressant use, use of other medications known to affect sleep, cigarette smoking, physical activity, total energy intake, alcohol intake, caffeine consumption, and Alternate Healthy Eating Index score.

^c^ BMI was modeled as less than 21, 21 to less than 23, 23 to less than 25, 25 to less than 27, 27 to less than 30, 30 to less than 33, 33 to less than 35, 35 to less than 40, or 40 or greater.

Sensitivity analyses supported our main findings. These included analyses with additional adjustment for hypothyroidism (eTable 5 in Supplement 1) and analyses using imputed values for missing data (eTable 6 in Supplement 1). A subgroup of women with sleep duration data collected repeatedly (2001, 2009, and 2017) during the follow-up period (n = 802) showed that a sleep duration of 6 hours per day was not associated with an increased risk of developing T2D (HR, 1.49; 95% CI, 0.87-2.55). This finding of no association was likely due to the smaller sample size (eTables 7 and 8 in Supplement 1).

## Discussion

In this prospective cohort study with a mean follow-up of 17.3 years, we found that a short sleep duration (≤6 hours per day) and regular or occasional snoring were associated with a higher risk of T2D among women with a history of GD. Additionally, we observed that more frequent snoring was associated with an unfavorable metabolic biomarker profile among women with a history of GD who did not have T2D at blood sample collection.

Women with a history of GD have a significantly higher risk of developing T2D compared with healthy controls.^9,10^ The latest consensus report by the ADA and the European Association for the Study of Diabetes recognizes sleep as an essential factor alongside traditional lifestyle factors, like diet and physical activity, in T2D management.^22^ However, research exploring the association between sleep and the development of T2D in women with GD remains sparse. Although this association has been established in the general population, the underlying glycemic status in women with GD differs significantly from that in the general population,^23^ potentially affecting the association between sleep and the development of T2D in this high-risk population. Women with GD often develop T2D faster and earlier than the general population, with 35% to 60% developing T2D within 10 years of GD diagnosis.^13,23^ Therefore, further investigation is needed to determine whether sleep plays a significant role in this high-risk group. Our study addressed this gap and revealed that a daily sleep duration of 7 to 8 hours was associated with the lowest risk of T2D among women with a history of GD. This is consistent with prior research in the general population, which also identified a U-shaped dose-response association between sleep duration and T2D risk, indicating 7 to 8 hours of sleep as the optimal duration for the lowest T2D risk.^5,24,25^ Some previous studies have similarly observed that a short sleep duration is associated with an increased incidence of T2D.^26,27^ For instance, an Australian prospective cohort study found that the risk of developing T2D was significantly higher in those who slept less than 6 hours compared with 7 hours per day, with an HR of 1.29 (95% CI, 1.08-1.53), while for those sleeping 10 or more hours per day, the HR was 1.00 (95% CI, 0.88-1.14).^27^ Another study using data from the National Health and Nutrition Examination Survey found that compared with the recommended sleep duration (>6 to <9 hours per day), short sleep duration (≤6 hours per day) was associated with higher risk of abnormal levels of HbA_1c_ and insulin in the general adult population.^28^

Although the exact molecular mechanisms are unclear, the observed association of short sleep duration and T2D is biologically plausible through the impacts of sympathetic nervous system activity and hormone changes on glucose metabolism.^29,30,31^ First, restricted sleep can lead to increased sympathetic nervous activity, which is crucial for the secretion of insulin and glucagon.^30^ Additionally, sleep restriction may activate the hypothalamic-pituitary-adrenal axis, leading to alterations in cortisol levels, which can then contribute to β-cell dysfunction and insulin resistance.^29,30,31^ Several randomized clinical trials with healthy volunteers have demonstrated the adverse effects of sleep restriction on glucose metabolism, resulting in a 16% to 29% reduction in insulin sensitivity compared with conditions of adequate rest.^32,33,34,35^ Second, sleep loss may alter the balance of appetite-regulating hormones like leptin and ghrelin, increasing food intake and stimulating the food reward system, which may result in weight gain and a higher BMI,^36^ subsequently contributing to insulin resistance.^30^ In our study, a significantly elevated risk of T2D persisted after adjustment for BMI, but HRs were lower. Third, other possible explanations for the association between short sleep duration and higher T2D risk may include decreased brain glucose utilization, an imbalance in sympathovagal activity, increased inflammatory markers, and adipocyte dysfunction.^30,37^

Our findings also revealed that higher risk of developing T2D was associated with the frequency of snoring among women with a history of GD. To our knowledge, there are no previous studies specifically exploring the relationship between snoring and the risk of developing T2D in this high-risk population. While research on snoring and T2D risk in the general population is emerging, the results have been inconsistent. Our findings are consistent with several prospective cohort studies that examined the association between snoring and the risk of T2D in the general population.^38,39^ The previous NHS study, which focused on female nurses aged 40 to 65 years, suggested that snoring might be independently associated with an increased risk of T2D, with a relative risk of 2.25 (95% CI, 1.91-2.66; *P* < .001 for trend).^38^ In the analyses stratified by BMI (<30 or ≥30), although the association between frequency of snoring and T2D risk was significant only among those with a BMI greater than 30, the point estimates of risk were similar across BMI strata, and the interaction term was not statistically significant. As such, there was no evidence to suggest that BMI significantly modified the associations.

Habitual snoring is a significant clinical feature and a surrogate marker of obstructive sleep apnea (OSA), which is a frequent comorbidity in patients with T2D.^40^ A meta-analysis reported that OSA was associated with a higher risk of T2D, with a pooled relative risk of 2.02 (95% CI, 1.57-2.61), which is comparable to that for traditional risk factors, such as having overweight and a family history of diabetes.^41^ Biologically, OSA may be linked to abnormal glucose metabolism and increased T2D risk through intermittent hypoxemia and sleep fragmentation.^40^ Hypoxia during OSA can trigger sympathetic nervous system activation, altering glucose levels and fat metabolism.^42^ Our biomarker results supported this biological mechanism linking habitual snoring with unfavorable glucose metabolism. We observed significant positive associations between snoring frequency and elevated levels of HbA_1c_, C-peptide, and insulin.

### Strengths and Limitations

To our knowledge, this is the first study to examine the association of sleep characteristics with both T2D risk and glucose metabolism biomarkers among women with a history of GD. The strengths of this study include its prospective cohort design, large sample size of women with GD, long-term follow-up period, and detailed information gathered on potential confounding factors. In addition, we evaluated the associations between sleep and glucose metabolism biomarkers among women with GD who had not developed T2D, thereby avoiding the potential impact of T2D treatments on the biomarker levels and possible bias due to reverse causation. Importantly, the results from both the metabolic biomarkers and the sensitivity analysis consistently aligned with those from our main analysis of incident T2D, thus substantially demonstrating the validity and robustness of our findings. Furthermore, participants in the NHSII are registered nurses, which helps to reduce potential confounding due to variations in educational attainment or access to health care.

We also acknowledge several potential limitations in our study. First, sleep characteristics were self-reported and were subject to measurement errors. Previous research showed that self-reported snoring had reasonable validity compared with whole-night recordings of body and breathing movements, with 93% of those classified as snorers by recordings also reporting habitual or occasional snoring.^43^ Due to the prospective nature of the study design, misclassifications of sleep characteristics, if any, would be nondifferential. As a result, the associations observed in our study might underestimate the true associations between sleep characteristics and T2D risk. Second, snoring and daytime sleepiness were reported only once at baseline, which may not accurately represent habitual sleep patterns. However, we conducted a sensitivity analysis of cumulative sleep duration with a small sample size and found consistent results, indicating a certain degree of robustness in our findings. Further prospective studies with time-updated information may be needed to validate our results. Third, our study population primarily consisted of White women in the US; thus, the generalization of our findings to other ethnic groups needs further evaluation. Fourth, we were unable to assess more detailed snoring characteristics (eg, duration and severity) or sleep behavior patterns (eg, weekend changes, social jetlag, or irregularity), which could provide a more comprehensive understanding of their associations with T2D.

## Conclusions

In this cohort study with a mean follow-up of 17.3 years encompassing a total of 42 155 person-years, we found that short sleep durations (≤6 hours per day) and frequent snoring (regular and occasional) were associated with an increased risk of T2D among women with a history of GD. In addition, snoring frequency was also associated with an unfavorable metabolic profile. These findings underscore the importance of sleep health, particularly for this high-risk population. Prevention strategies for progression from GD to T2D should incorporate sleep health, emphasizing the monitoring of sleep duration and snoring.

## Notes

Supplement 1.
eMethods.

eReferences.
**eTable 1.** Association of Sleep Characteristics and Risk of T2D Among Women With a History of GD, Stratified by Different Lifestyle Factors, Nurses’ Health Study II **eTable 2.** Joint Association of Sleep Duration and Snore Frequency With Risk of T2D Among Women With a History of GD, Nurses’ Health Study II **eTable 3.** Joint Association of Sleep Duration and Daytime Sleepiness With Risk of T2D Among Women With a History of GD, Nurses’ Health Study II **eTable 4.** Joint Association of Daytime Sleepiness and Snore Frequency With Risk of T2D Among Women With a History of GD, Nurses’ Health Study II **eTable 5.** Association of Sleep Characteristics With Risk of T2D Among Women With a History of GD, Further Adjusted for Hypothyroidism, Nurses’ Health Study II **eTable 6.** Association of Sleep Characteristics With Risk of T2D Among Women With a History of GD, Using Multiple Imputation for Covariates With Missing Values, Nurses’ Health Study II **eTable 7.** Age-Standardized Baseline Characteristics of Women With a History of GD and Available Time-Updated Sleep Data According to Their Sleep Duration, Nurses’ Health Study II **eTable 8.** Association of Sleep Duration and Risk of T2D Among Women With a History of GD and Available Time-Updated Sleep Data, Nurses’ Health Study II

Supplement 2.
Data Sharing Statement
