# Supplementary material for: Sleep Characteristics and Long-Term Risk of Type 2 Diabetes Among Women With Gestational Diabetes
Source: JAMA Netw Open. 2025 Mar 5;8(3):e250142. doi: 10.1001/jamanetworkopen.2025.0142 (PMC11883505; doi:10.1001/jamanetworkopen.2025.0142)
Supplement: Supplement 1. — eMethods. eReferences. eTable 1. Association of Sleep Characteristics and Risk of T2D Among Women With a History of GD, Stratified by Different Lifestyle Factors, Nurses’ Health Study II eTable 2. Joint Association of Sleep Duration and Snore Frequency With Risk of T2D Among Women With a History of GD, Nurses’ Health Study II eTable 3. Joint Association of Sleep Duration and Daytime Sleepiness With Risk of T2D Among Women With a History of GD, Nurses’ Health Study II eTable 4. Joint Association of Daytime Sleepiness and Snore Frequency With Risk of T2D Among Women With a History of GD, Nurses’ Health Study II eTable 5. Association of Sleep Characteristics With Risk of T2D Among Women With a History of GD, Further Adjusted for Hypothyroidism, Nurses’ Health Study II eTable 6. Association of Sleep Characteristics With Risk of T2D Among Women With a History of GD, Using Multiple Imputation for Covariates With Missing Values, Nurses’ Health Study II eTable 7. Age-Standardized Baseline Characteristics of Women With a History of GD and Available Time-Updated Sleep Data According to Their Sleep Duration, Nurses’ Health Study II eTable 8. Association of Sleep Duration and Risk of T2D Among Women With a History of GD and Available Time-Updated Sleep Data, Nurses’ Health Study II [file jamanetwopen-e250142-s001.pdf]

Supplementary Online Content

Yin X, Bao W, Ley SH, et al. Sleep characteristics and long-term risk of type 2 diabetes for women with gestational diabetes. *JAMA Netw Open*. 2025;8(3):e250142. doi:10.1001/jamanetworkopen.2025.0142

eMethods.

eReferences.

eTable 1. Association of Sleep Characteristics and Risk of T2D Among Women With a History of GD, Stratified by Different Lifestyle Factors, Nurses' Health Study II

eTable 2. Joint Association of Sleep Duration and Snore Frequency With Risk of T2D Among Women With a History of GD, Nurses' Health Study II

eTable 3. Joint Association of Sleep Duration and Daytime Sleepiness With Risk of T2D Among Women With a History of GD, Nurses' Health Study II

eTable 4. Joint Association of Daytime Sleepiness and Snore Frequency With Risk of T2D Among Women With a History of GD, Nurses' Health Study II

eTable 5. Association of Sleep Characteristics With Risk of T2D Among Women With a History of GD, Further Adjusted for Hypothyroidism, Nurses' Health Study II

eTable 6. Association of Sleep Characteristics With Risk of T2D Among Women With a History of GD, Using Multiple Imputation for Covariates With Missing Values, Nurses' Health Study II

eTable 7. Age-Standardized Baseline Characteristics of Women With a History of GD and Available Time-Updated Sleep Data According to Their Sleep Duration, Nurses' Health Study II

eTable 8. Association of Sleep Duration and Risk of T2D Among Women With a History of GD and Available Time-Updated Sleep Data, Nurses' Health Study II

This supplementary material has been provided by the authors to give readers additional information about their work.

© 2025 Yin X et al. *JAMA Network Open*.

eMethods.

Study Population

In this study, we included women who reported a history of GD and answered questions on sleep characteristics in the 2001 questionnaire, which served as the baseline for follow-up. GD cases were identified from 1989 to 2001 in the NHSII cohort. The 2001 questionnaire was the last to include questions regarding GD, as most NHSII participants had passed reproductive age. In a prior validation study, 94% of self-reported GD cases were confirmed by medical records [4]. In a random sample of parous women without GD, 83% reported a glucose screening test during pregnancy and 100% reported frequent prenatal urine screening, suggesting a high level of GD surveillance in this cohort [4]. At baseline, we excluded women from the analysis if they: (1) had a history of type 1 diabetes, multiple gestation pregnancies (twins or multiple births), or missing birth dates; (2) had a history of T2D, cardiovascular disease (myocardial infarction or stroke), or cancer before 2001 when sleep characteristics were reported; (3) were diagnosed with T2D prior to GD; or (4) had missing data on sleep-related factors; or (5) did not report having a history of GD. The final dataset consisted of 2,891 participants with a history of GD. These women were followed biennially until June 2021.

Assessment of Exposure Variables

Sleep characteristics were self-reported only once in the 2001 baseline questionnaire. Snoring frequency response options ranged from almost never, occasionally (1-2 nights/week), a few nights a week (3-4 nights/week), most nights (5-6 nights/week), to every night. We recategorized these into three groups: almost never (0 nights/week), occasionally (1-2 nights/week), and regularly (≥ 3 nights/week). Participants reported their average sleep duration with choices of <5, 5, 6, 7, 8, 9, and ≥10 hours, validated against a week of sleep diaries from a similar cohort of U.S. nurses [5]. We categorized sleep duration into ≤6, 7-8, and ≥9 hours for our main analysis. Daytime sleepiness was assessed with the question, "On average, how often are your daily activities affected because you are sleepy during the day?" Responses ranged from never, rarely, 1-3 days/week, 4-6 days/week, to every day. We categorized these into three groups: rarely or never (0 days/week), 1-3 days/week, and ≥ 4 days/week.

Ascertainment of Outcome Variables

Participants reporting physician-diagnosed T2D on each biennial questionnaire were mailed a supplementary questionnaire regarding symptoms, diagnostic tests, and hypoglycemic therapy to confirm self-reported diagnoses. Confirmed diabetes required at least one of the following reported on the supplementary questionnaire according to the American Diabetes Association (ADA) criteria [6]: (1) one or more classic symptoms (excessive thirst, polyuria, weight loss, hunger, pruritus, or coma) plus elevated glucose levels (fasting plasma glucose concentration ≥ 7.0 mmol/L or random plasma glucose ≥ 11.1 mmol/L); or (2) no symptoms reported but two or more elevated plasma glucose concentrations on more than one occasion

© 2025 Yin X et al. *JAMA Network Open*.

(fasting ≥ 7.0 mmol/L, random ≥ 11.1 mmol/L, 2-hour oral glucose tolerance test ≥ 11.1 mmol/L); or (3) treatment with insulin or oral hypoglycemic agent. A high accuracy rate of 98% [7] and a low frequency of under-reporting at 0.5% [8] were observed when comparing our classification with reviews of medical records.

Assessment of Biomarkers

In 2012-2014, 3,667 women with a history of GD from the NHSII cohort were invited to the DWH Study for the questionnaire and biospecimen collection. Detailed study design and methods can be found in previous publications [1,2]. As previously documented, the characteristics of participants who provided blood samples were comparable to those who did not provide blood samples [1,2]. Blood samples were sent by overnight shipping to an accredited laboratory at the University of Minnesota.

In this study, we assessed HbA1c, a diagnostic criterion for T2D, as well as insulin and C-peptide levels, which serve as biomarkers for blood glycemic metabolism [9]. The HbA1c measurements were performed using a non-porous ion exchange high performance liquid chromatography (HPLC) assay (Tosoh Automated Analyzer HLC-723G8, Tosoh Bioscience, Inc., San Francisco, CA). The coefficient of variation (CV) for HbA1c measurements was <1.2%. C-peptide concentration was measured utilizing the C-peptide micro-ELISA method (Quansys Biosciences). Insulin concentrations were assessed using the Cobas 6000 chemistry analyzer (Roche Diagnostics). The inter-assay CVs ranged from 1.2% to 10.0% for all assays.

In examining the associations between sleep characteristics and glycemic metabolism biomarkers, we excluded women who (1) received a T2D diagnosis prior to the time of blood collection, (2) had an HbA1c level of ≥ 6.5% at blood collection as suspected undiagnosed diabetes, and (3) lacked all glucose metabolism biomarkers of interest (i.e. HbA1c, C-peptide, and insulin). A total of 527 women with a history of GD who provided both fasting blood samples and sleep characteristics were included in our final biomarker analysis.

Assessment of Covariates

In NHSII, participants provided updates on demographics, lifestyle, and health conditions every 2-4 years. For covariates except race/ethnicity and diet-related factors, data collected in the 2001 questionnaire were used, aligning with the year that data on sleep were collected. Race/ethnicity was reported in the 1989 baseline questionnaire of NHSII. Dietary information was assessed every 4 years since 1991 using a semi-quantitative food frequency questionnaire (FFQ), which has been extensively validated [10]. We utilized dietary information from the 1999 FFQ because it was the one closest to the baseline (2001). Missing covariate data were carried forward from preceding questionnaires where available. Values from before GD diagnosis were not carried forward.

© 2025 Yin X et al. *JAMA Network Open*.

Parity was defined as the number of pregnancies lasting over 6 months. Body mass index (BMI) was calculated as weight in kilograms divided by height in meters squared (kg/m^2). Shift work status was categorized by whether the women had ever worked rotating night shifts before 2001. Depressive symptoms were assessed using the SF-36 five-item Mental Health Index (MHI-5) [11], with scores of 0-52 indicating depression [12]. Regular use of antidepressants and other medications affecting sleep (beta-blockers, oral steroids, and minor tranquilizers) over the past 2 years was collected. Respiratory illnesses included asthma, emphysema, chronic bronchitis, or pneumonia. Diet quality was assessed with the Alternate Healthy Eating Index 2010 (AHEI) from the FFQ [13]. Unusual FFQ responses, including more than 70 items left blank or total energy intake falling below 500 or exceeding 3500 kcal/day, were addressed using previous cycle values. Caffeine consumption, alcohol intake, and total energy intake were also derived from the FFQ. Physical activity was measured in metabolic equivalent task (MET)-hours per week based on recreational activity frequency, validated against detailed activity diaries [14].

Statistical Analysis

Data were analyzed from November 2023 to August 2024. Follow-up time was calculated from the 2001 baseline to T2D diagnosis, death, last biennial questionnaire response, or June 2021, whichever came first. We used Cox proportional hazards models to estimate hazard ratios (HRs) and 95% confidence intervals (CIs) for the association between baseline sleep characteristics and the risk of T2D. All Cox proportional hazards models were stratified by age (in months) and calendar time. We conducted a likelihood ratio test comparing models with and without the interaction term between exposure and age (as a proxy for calendar time; <45 years or ≥45 years (i.e., median)). The non-significant likelihood ratio tests indicated no violation of the proportional hazards assumption. We adjusted for potential confounding factors, including parity (1, 2, ≥3), race/ethnicity (White or non-White), family history of diabetes (yes or no), oral contraceptive use (never, past, current user), menopausal status (premenopausal or postmenopausal), cigarette smoking (never, past, current smoker), physician-diagnosed respiratory illnesses including asthma, emphysema, chronic bronchitis, and pneumonia (yes or no), depression (yes or no), use of antidepressants or other medications known to affect sleep (never or ever), caffeine consumption (quartiles), alcohol intake (quartiles), total energy intake (quartiles), diet quality (i.e., AHEI score, in quartiles), physical activity (MET-hours/week, in quartiles) and BMI (as <21, 21 to <23, 23 to <25, 25 to <27, 27 to <30, 30 to <33, 33 to <35, 35 to <40, or ≥40 kg/m^2). We investigated potential non-linear associations between sleep durations and the risk of T2D incidence using restricted cubic splines with three knots, excluding values exceeding 2.5% and 97.5% percentiles [15]. Joint associations between sleep characteristics were also tested. Additionally, we conducted stratified analyses on T2D risk factors, including age, obesity status, diet quality (AHEI score), alcohol intake, physical activity, and family history of diabetes, to examine potential differences across various strata of these risk factors.

© 2025 Yin X et al. *JAMA Network Open*.

We tested each biomarker for normality, and applied log-transformation where distributions were skewed. Generalized linear models were used to estimate the least-squares means of metabolic biomarker levels across categories of sleep characteristics. To test for linear trends across these categories, we assigned the median value to each category and incorporated this continuous variable into the models.

In addition to 2001 baseline sleep characteristics, we conducted a sensitivity analysis among a sub-group of women (n=802) who provided updated data on sleep duration in 2001, 2009, and 2017. Snoring and daytime sleepiness were not collected regularly during the follow-up period. For these 802 individuals, we used cumulative average sleep data from the first follow-up cycle (2001 baseline) through the time of censoring events. Covariates were also time-updated. Time-updated cumulative averages for diet-related variables (including caffeine consumption, alcohol intake, total energy intake, and AHEI score) were calculated using the FFQs from 1999, 2007, and 2015, which were the closest to the sleep data collection periods. All statistical analyses were performed using SAS software (version 9.3; SAS Institute Inc.). A two-sided $P < 0.05$ was considered statistically significant.

© 2025 Yin X et al. *JAMA Network Open*.

eReferences

1. Zhang C, Hu FB, Olsen SF, et al. Rationale, design, and method of the Diabetes & Women's Health study - A study of long-term health implications of glucose intolerance in pregnancy and their determinants. *Acta obstetricia et gynecologica Scandinavica*. May 14 2014;93(11):1123-30. doi:10.1111/aogs.12425
2. Zhang C, Olsen SF, Hinkle SN, et al. Diabetes & Women's Health (DWH) Study: an observational study of long-term health consequences of gestational diabetes, their determinants and underlying mechanisms in the USA and Denmark. *BMJ Open*. May 1 2019;9(4):e025517. doi:10.1136/bmjopen-2018-025517
3. Bao Y, Bertoia ML, Lenart EB, et al. Origin, Methods, and Evolution of the Three Nurses' Health Studies. *Am J Public Health*. Sep 2016;106(9):1573-81. doi:10.2105/ajph.2016.303338
4. Solomon CG, Willett WC, Carey VJ, et al. A prospective study of pregravid determinants of gestational diabetes mellitus. *JAMA*. Oct 1 1997;278(13):1078-83.
5. Patel SR, Ayas NT, Malhotra MR, et al. A prospective study of sleep duration and mortality risk in women. Research Support, U.S. Gov't, P.H.S. *Sleep*. May 1 2004;27(3):440-4.
6. Expert Committee on the Diagnosis and Classification of Diabetes Mellitus. Report of the Expert Committee on the Diagnosis and Classification of Diabetes Mellitus. *Diabetes Care*. Jul 1997;20(7):1183-97.
7. Manson JE, Rimm EB, Stampfer MJ, et al. Physical activity and incidence of non-insulin-dependent diabetes mellitus in women. *Lancet*. Sep 28 1991;338(8770):774-8. doi:10.1016/0140-6736(91)90664-b
8. Field AE, Coakley EH, Must A, et al. Impact of overweight on the risk of developing common chronic diseases during a 10-year period. *Arch Intern Med*. Jul 9 2001;161(13):1581-6. doi:10.1001/archinte.161.13.1581
9. Nathan DM, Turgeon H, Regan S. Relationship between glycated haemoglobin levels and mean glucose levels over time. *Diabetologia*. Nov 2007;50(11):2239-2244. doi:10.1007/s00125-007-0803-0
10. Willett WC, Reynolds RD, Cottrell-Hoehner S, Sampson L, Browne ML. Validation of a semi-quantitative food frequency questionnaire: comparison with a 1-year diet record. *J Am Diet Assoc*. Jan 1987;87(1):43-7.
11. Friedman B, Heisel M, Delavan R. Validity of the SF-36 five-item Mental Health Index for major depression in functionally impaired, community-dwelling elderly patients. *Journal of the American Geriatrics Society*. Nov 2005;53(11):1978-85. doi:10.1111/j.1532-5415.2005.00469.x
12. Arroyo C, Hu FB, Ryan LM, et al. Depressive symptoms and risk of type 2 diabetes in women. *Diabetes Care*. Jan 2004;27(1):129-33.
13. Chiuve SE, Fung TT, Rimm EB, et al. Alternative dietary indices both strongly predict risk of chronic disease. Research Support, N.I.H., Extramural
Research Support, Non-U.S. Gov't. *The Journal of nutrition*. Jun 2012;142(6):1009-18. doi:10.3945/jn.111.157222

© 2025 Yin X et al. *JAMA Network Open*.

14. Wolf AM, Hunter DJ, Colditz GA, et al. Reproducibility and validity of a self-administered physical activity questionnaire. *International journal of epidemiology*. Oct 1994;23(5):991-9.

15. Harrell F. *Regression Modeling Strategies: With Applications to Linear Models, Logistic and Ordinal Regression, and Survival Analysis*. 2015.

© 2025 Yin X et al. *JAMA Network Open*.

eTable 1. Association of sleep characteristics and risk of T2D among women with a history of GD, stratified by different lifestyle factors, Nurses' Health Study II

**Frequency of snoring**	**Almost never**	**Occasionally**	**Regularly**
Age			
<45 years (median)	1.00 (reference)	1.47 (0.98-2.19)	1.41 (0.91-2.18)
≥45 years (median)	1.00 (reference)	**1.70 (1.17-2.47)**	**1.90 (1.28-2.80)**
Family history of diabetes			
No	1.00 (reference)	1.49 (0.96-2.32)	**1.61 (1.01-2.56)**
Yes	1.00 (reference)	**1.64 (1.08-2.48)**	**1.67 (1.08-2.58)**
BMI [a]			
<30 kg/m²	1.00 (reference)	**1.50 (1.03-2.19)**	**1.70 (1.11-2.59)**
≥30 kg/m²	1.00 (reference)	1.44 (0.86-2.41)	1.50 (0.89-2.53)
Physical activity (MET-hr/wk)			
<10.2 MET-hr/wk (median)	1.00 (reference)	1.26 (0.86-1.87)	1.37 (0.91-2.06)
≥10.2 MET-hr/wk (median)	1.00 (reference)	**1.82 (1.12-2.94)**	**2.01 (1.20-3.37)**
AHEI score			
<45.259 (<median)	1.00 (reference)	1.25 (0.85-1.83)	1.23 (0.82-1.84)
≥45.259 (≥median)	1.00 (reference)	**1.69 (1.06-2.67)**	**2.15 (1.27-3.64)**
**Average sleep duration**	**≤ 6 hours/day**	**7-8 hours/day**	**≥ 9 hours/day**
Age			
<45 years (median)	**1.51 (1.06-2.16)**	1.00 (reference)	1.37 (0.73-2.58)
≥45 years (median)	1.16 (0.86-1.56)	1.00 (reference)	0.87 (0.45-1.67)
Family history of diabetes			
No	1.22 (0.85-1.76)	1.00 (reference)	1.35 (0.64-2.81)
Yes	**1.57 (1.09-2.25)**	1.00 (reference)	0.71 (0.37-1.39)
BMI [a]			
<30 kg/m²	1.15 (0.81-1.63)	1.00 (reference)	1.31 (0.70-2.46)
≥30 kg/m²	**1.52 (1.05-2.21)**	1.00 (reference)	0.75 (0.33-1.71)
Physical activity (MET-hr/wk)			
<10.2 MET-hr/wk (median)	1.17 (0.85-1.61)	1.00 (reference)	0.90 (0.44-1.83)
≥10.2 MET-hr/wk (median)	**1.94 (1.27-2.96)**	1.00 (reference)	**2.16 (1.02-4.55)**
AHEI score			
<45.259 (<median)	1.39 (0.99-1.93)	1.00 (reference)	1.45 (0.76-2.77)

© 2025 Yin X et al. *JAMA Network Open*.

≥45.259 (≥median)	1.36 (0.92-2.00)	1.00 (reference)	0.77 (0.33-1.77)

Data were HRs (95% CIs) unless noted otherwise. Cox proportional hazards regression models were stratified by age (months) and calendar time and adjusted for parity (1, 2, ≥3), race/ethnicity (White/non-White), family history of diabetes (yes or no), oral contraceptive use (never, past, current user), menopausal status (premenopausal or postmenopausal), ever shift work (yes, no), respiratory illnesses (yes, no), depression (yes, no), antidepressants use (yes, no), use of other medications known to affect sleep including beta-blockers, oral steroids, and minor tranquilizers (yes, no), cigarette smoking (current, former, never), physical activity (MET-hours/week, in quartiles), total energy intake (quartiles), alcohol intake (quartiles), caffeine consumption (quartiles), AHEI score (quartiles), and BMI (<21, 21 to <23, 23 to <25, 25 to <27, 27 to <30, 30 to <33, 33 to <35, 35 to <40, or ≥40 kg/m2). Bold values refer to statistically significant results with *P*<0.05.

[a] BMI was further adjusted as continuous variable (kg/m^2).

© 2025 Yin X et al. *JAMA Network Open*.

eTable 2. Joint association between sleep duration and snore frequency on the risk of T2D among women with a history of GD, Nurses' Health Study II

**Snore**	**Sleep duration**		
	**≤ 6 hours/day**	**7-8 hours/day**	**≥ 9 hours/day**
**Almost never**			
Number of participants	321	672	54
T2D cases (n)	37	75	8
HR (95%CI)	0.96 (0.61-1.52)	1.00 (reference)	1.04 (0.39-2.74)
**Occasionally**			
Number of participants	336	660	43
T2D cases (n)	77	130	9
HR (95%CI)	**2.04 (1.39-2.99)**	1.34 (0.96-1.87)	1.27 (0.58-2.80)
**Regularly**			
Number of participants	253	498	54
T2D cases (n)	86	124	17
HR (95%CI)	**2.06 (1.38-3.07)**	**1.43 (1.01-2.02)**	1.52 (0.77-2.97)

Data were HRs (95% CIs) unless noted otherwise. Cox proportional hazards regression models were stratified by age (months) and calendar time and adjusted for parity (1, 2, ≥3), race/ethnicity (White/non-White), family history of diabetes (yes or no), oral contraceptive use (never, past, current user), menopausal status (premenopausal or postmenopausal), ever shift work (yes, no), respiratory illnesses (yes, no), depression (yes, no), antidepressants use (yes, no), use of other medications known to affect sleep including beta-blockers, oral steroids, and minor tranquilizers (yes, no), cigarette smoking (current, former, never), physical activity (MET-hours/week, in quartiles), total energy intake (quartiles), alcohol intake (quartiles), caffeine consumption (quartiles), AHEI score (quartiles) and BMI (<21, 21 to <23, 23 to <25, 25 to <27, 27 to <30, 30 to <33, 33 to <35, 35 to <40, or ≥40 kg/m^2). Bold values refer to statistically significant results with *P*<0.05.

© 2025 Yin X et al. *JAMA Network Open*.

eTable 3. Joint association between sleep duration and daytime sleepiness on the risk of T2D among women with a history of GD, Nurses' Health Study II

Frequency of daytime sleepiness	Sleep duration		
	**≤ 6 hours/day**	**7-8 hours/day**	**≥ 9 hours/day**
**Rarely or never**			
Number of participants	469	1173	82
T2D cases (n)	93	200	15
HR (95%CI)	1.20 (0.89-1.62)	1.00 (reference)	0.97 (0.52-1.81)
**1-3 days/week**			
Number of participants	253	436	29
T2D cases (n)	52	80	11
HR (95%CI)	1.24 (0.85-1.81)	0.95 (0.70-1.30)	1.47 (0.62-3.45)
**≥ 4 days/week**			
Number of participants	188	221	40
T2D cases (n)	55	49	8
HR (95%CI)	**1.54 (1.04-2.30)**	0.90 (0.61-1.33)	0.77 (0.32-1.84)

Data were HRs (95% CIs) unless noted otherwise. Cox proportional hazards regression models were stratified by age (months) and calendar time and adjusted for parity (1, 2, ≥3), race/ethnicity (White/non-White), family history of diabetes (yes or no), oral contraceptive use (never, past, current user), menopausal status (premenopausal or postmenopausal), ever shift work (yes, no), respiratory illnesses (yes, no), depression (yes, no), antidepressants use (yes, no), use of other medications known to affect sleep including beta-blockers, oral steroids, and minor tranquilizers (yes, no), cigarette smoking (current, former, never), physical activity (MET-hours/week, in quartiles), total energy intake (quartiles), alcohol intake (quartiles), caffeine consumption (quartiles), AHEI score (quartiles), and BMI (<21, 21 to <23, 23 to <25, 25 to <27, 27 to <30, 30 to <33, 33 to <35, 35 to <40, or ≥40 kg/m2). Bold values refer to statistically significant results with *P*<0.05.

© 2025 Yin X et al. *JAMA Network Open*.

eTable 4. Joint association between daytime sleepiness and snore frequency on the risk of T2D among women with a history of GD, Nurses' Health Study II

Snore	Daytime sleepiness		
	**Rarely or never**	**1-3 days/week**	**≥ 4 days/week**
**Almost never**			
Number of participants	703	235	109
T2D cases (n)	78	27	15
HR (95%CI)	1.00 (reference)	0.94 (0.57-1.55)	1.16 (0.59-2.28)
**Occasionally**			
Number of participants	614	262	163
T2D cases (n)	132	46	38
HR (95%CI)	**1.62 (1.16-2.25)**	**1.52 (1.00-2.33)**	1.31 (0.81-2.14)
**Regularly**			
Number of participants	407	221	177
T2D cases (n)	98	70	59
HR (95%CI)	**1.47 (1.02-2.12)**	**1.70 (1.12-2.58)**	**1.78 (1.14-2.77)**

Data were HRs (95% CIs) unless noted otherwise. Cox proportional hazards regression models were stratified by age (months) and calendar time and adjusted for parity (1, 2, ≥3), race/ethnicity (White/non-White), family history of diabetes (yes or no), oral contraceptive use (never, past, current user), menopausal status (premenopausal or postmenopausal), ever shift work (yes, no), respiratory illnesses (yes, no), depression (yes, no), antidepressants use (yes, no), use of other medications known to affect sleep including beta-blockers, oral steroids, and minor tranquilizers (yes, no), cigarette smoking (current, former, never), physical activity (MET-hours/week, in quartiles), total energy intake (quartiles), alcohol intake (quartiles), caffeine consumption (quartiles), AHEI score (quartiles), and BMI (<21, 21 to <23, 23 to <25, 25 to <27, 27 to <30, 30 to <33, 33 to <35, 35 to <40, or ≥40 kg/m2). Bold values refer to statistically significant results with *P*<0.05.

© 2025 Yin X et al. *JAMA Network Open*.

eTable 5. Association of sleep characteristics with risk of T2D among women with a history of GD, further adjusted for hypothyroidism, Nurses' Health Study II

	Frequency of snoring			
	Almost never	Occasionally	Regularly	*P* -trend
Full adjusted model [a]	1.00 (reference)	**1.54 (1.18-2.02)**	**1.61 (1.21-2.13)**	0.01
+ Hypothyroidism [b]	1.00 (reference)	**1.54 (1.18-2.01)**	**1.60 (1.21-2.13)**	0.01
	Average sleep duration			
	≤ 6 hours/day	7-8 hours/day	≥ 9 hours/day	*P* -trend [c]
Full adjusted model [a]	**1.32 (1.06-1.64)**	1.00 (reference)	1.03 (0.66-1.61)	-
+ Hypothyroidism [b]	**1.32 (1.06-1.65)**	1.00 (reference)	1.04 (0.66-1.61)	-
	Frequency of daytime sleepiness			
	Rarely or never	1-3 days/week	≥ 4 days/week	*P* -trend
Full adjusted model [a]	1.00 (reference)	1.02 (0.80-1.30)	1.06 (0.80-1.41)	0.68
+ Hypothyroidism [b]	1.00 (reference)	1.02 (0.80-1.30)	1.06 (0.80-1.41)	0.68

Data were HRs (95% CIs) unless noted otherwise. Bold values refer to statistically significant results with $P<0.05$.

[a] Cox proportional hazards regression models were stratified by age (months) and calendar time and adjusted for parity (1, 2, ≥3), race/ethnicity (White/non-White), family history of diabetes (yes, no), oral contraceptive use (never, past, current user), menopausal status (premenopausal or postmenopausal), ever shift work (yes, no), respiratory illnesses (yes, no), depression (yes, no), antidepressants use (yes, no), use of other medications known to affect sleep including beta-blockers, oral steroids, and minor tranquilizers (yes, no), cigarette smoking (current, former, never), physical activity (MET-hours/week, in quartiles), total energy intake (quartiles), alcohol intake (quartiles), caffeine consumption (quartiles), AHEI score (quartiles), and BMI (<21, 21 to <23, 23 to <25, 25 to <27, 27 to <30, 30 to <33, 33 to <35, 35 to <40, or ≥40 kg/m^2).

[b] Further adjusted for hypothyroidism (yes, no).

[c] *P* for trend was not tested due to the non-linear association observed between sleep duration and the risk of T2D.

© 2025 Yin X et al. *JAMA Network Open*.

eTable 6. Association of sleep characteristics with risk of T2D among women with a history of GD, using multiple imputation for covariates with missing values, Nurses' Health Study II

	Frequency of snoring			
	Almost never	Occasionally	Regularly	*P* -trend
Full adjusted model [a]	1.00 (reference)	**1.54 (1.20-1.99)**	**1.66 (1.27-2.16)**	0.002
	Average sleep duration			
	≤ 6 hours/day	7-8 hours/day	≥ 9 hours/day	*P* -trend [c]
Full adjusted model [a]	**1.37 (1.11-1.69)**	1.00 (reference)	1.03 (0.68-1.57)	-
	Frequency of daytime sleepiness			
	Rarely or never	1-3 days/week	≥ 4 days/week	*P* -trend
Full adjusted model [a]	1.00 (reference)	1.02 (0.81-1.29)	1.12 (0.86-1.47)	0.41

Data were HRs (95% CIs) unless noted otherwise. Bold values refer to statistically significant results with $P<0.05$.

[a] Cox proportional hazards regression models were stratified by age (months) and calendar time and adjusted for parity (1, 2, ≥3), race/ethnicity (White/non-White), family history of diabetes (yes, no), oral contraceptive use (never, past, current user), menopausal status (premenopausal or postmenopausal), ever shift work (yes, no), respiratory illnesses (yes, no), depression (yes, no), antidepressants use (yes, no), use of other medications known to affect sleep including beta-blockers, oral steroids, and minor tranquilizers (yes, no), cigarette smoking (current, former, never), physical activity (MET-hours/week, in quartiles), total energy intake (quartiles), alcohol intake (quartiles), caffeine consumption (quartiles), AHEI score (quartiles), and BMI (<21, 21 to <23, 23 to <25, 25 to <27, 27 to <30, 30 to <33, 33 to <35, 35 to <40, or ≥40 kg/m^2).

[c] *P* for trend was not tested due to the non-linear association observed between sleep duration and the risk of T2D.

© 2025 Yin X et al. *JAMA Network Open*.

eTable 7. Age-standardized baseline characteristics of women with a history of GD according to their sleep durations (hours/day), among women with available time-updated sleep data, Nurses' Health Study II (N=802)

	**Average sleep duration (hours/day)**		
	**≤ 6**	**7-8**	**≥ 9**
Number of participants	242	515	45
Age (years), mean (SD)	46.3 (4.6)	46.3 (4.8)	45.7 (4.6)
Age (years) at first report of GD, mean (SD)	32.8 (5.5)	33.1 (5.1)	33.2 (4.8)
Race, White, N (%)	89.9 (217)	93.8 (483)	91.3 (41)
Family history of diabetes, N (%)	54.0 (131)	48.9 (252)	49.7 (22)
Parity, median (IQR)	2 (2,3)	2 (2,3)	2 (2,3)
BMI at baseline, kg/m²	28.8 (6.7)	27.3 (6.2)	27.7 (3.5)
Oral contraceptives use, N (%)			
- Never use	12.2 (29)	13.6 (70)	8.3 (4)
- Past user	82.0 (196)	79.1 (406)	85.5 (38)
- Current user	5.8 (14)	7.3 (38)	6.2 (3)
Postmenopausal, N (%)	17.2 (41)	17.9 (92)	24.5 (11)
Ever shift worker, N (%)	33.0 (78)	27.9 (141)	32.3 (14)
Respiratory illness, N (%) [a]	19.6 (47)	15.8 (82)	22.4 (10)
Depression, N (%)	17.9 (43)	12.8 (66)	28.1 (13)
Antidepressants, N (%)	19.6 (47)	20.2 (104)	36.7 (17)
Current use of other medications known to affect sleep			
- Beta-blockers, N (%)	4.3 (10)	5.3 (27)	8.2 (4)
- Steroids, N (%)	3.4 (8)	2.0 (10)	4.3 (2)
- Minor tranquilizers, N (%)	5.2 (13)	3.2 (17)	1.5 (1)
Physical activity (MET-hr/wk), mean (SD) [b]	17.8 (17.5)	17.9 (20.1)	18.2 (10.4)
AHEI score, mean (SD)	50 (10.5)	51.2 (11.4)	47.6 (5.8)
Alcohol intake (g/d), mean (SD)	3.2 (5.3)	3.3 (5.4)	1.4 (2)
Caffeine intake (mg/d), mean (SD)	236 (202)	213 (189)	196 (109)
Total calories (kcal/d), mean (SD)	1891 (551)	1919 (572)	1973 (316)

Abbreviations: GD, gestational diabetes; AHEI: Alternate Healthy Eating Index with alcohol component removed; BMI: body mass index; MET, metabolic equivalents; SD, standard deviation. IQR, interquartile range.

[a] Including asthma, emphysema, chronic bronchitis, and pneumonia.

© 2025 Yin X et al. *JAMA Network Open*.

[b] MET values were calculated from the sum of weekly leisure-time moderate- or vigorous-intensity physical activities. 7.5 MET-h/week is equivalent to 150 min/week of moderate intensity physical activity or 75 min/week of vigorous intensity physical activity.

© 2025 Yin X et al. *JAMA Network Open*.

eTable 8. Association of sleep durations and risk of T2D among women with a history of GD and available time-updated sleep data, Nurses' Health Study II (N=802)

**Average sleep duration**	**≤ 6 hours/day**	**7-8 hours/day**	**≥ 9 hours/day**
Number of participants	248	490	64
T2D cases (n)	56	96	15
Person-years	3291.0	7721.5	852.2
No of cases/1000-person years	17.0	12.4	17.6
Age-adjusted model	**1.58 (1.06-2.35)**	1.00 (reference)	1.52 (0.78-2.99)
Multivariable model [a]	1.52 (0.96-2.42)	1.00 (reference)	1.40 (0.66-2.99)
Multivariable model + BMI [b]	1.49 (0.87-2.55)	1.00 (reference)	0.91 (0.38-2.17)

Data were HRs (95% CIs) unless noted otherwise. Bold values refer to statistically significant results with P<0.05.

[a] Cox proportional hazards regression models were stratified by age (months) and calendar time and adjusted for parity (1, 2, ≥3), race/ethnicity (White/non-White), family history of diabetes (yes, no), oral contraceptive use (never, past, current user), menopausal status (premenopausal or postmenopausal), ever shift work (yes, no), respiratory illnesses (yes, no), depression (yes, no), antidepressants use (yes, no), use of other medications known to affect sleep including beta-blockers, oral steroids, and minor tranquilizers (yes, no), cigarette smoking (current, former, never), physical activity (MET-hours/week, in quartiles), total energy intake (quartiles), alcohol intake (quartiles), caffeine consumption (quartiles), and AHEI score (quartiles).

[b] BMI was modeled as <21, 21 to <23, 23 to <25, 25 to <27, 27 to <30, 30 to <33, 33 to <35, 35 to <40, or ≥40 kg/m^2.

© 2025 Yin X et al. *JAMA Network Open*.
