# Supplementary material for: Sleep Characteristics and Long-Term Risk of Type 2 Diabetes Among Women With Gestational Diabetes
Source: JAMA Netw Open. 2025 Mar 5;8(3):e250142. doi: 10.1001/jamanetworkopen.2025.0142 (PMC11883505; doi:10.1001/jamanetworkopen.2025.0142)
Supplement: Supplement 2. — Data Sharing Statement [file jamanetwopen-e250142-s002.pdf]

Data Sharing Statement

Yin. Sleep Characteristics and Long-Term Risk of Type 2 Diabetes Among Women With Gestational Diabetes. *JAMA Netw Open*. Published March 05, 2025.
doi:10.1001/jamanetworkopen.2025.0142

Data
Data available: No

Additional Information
Explanation for why data not available: Statistical analysis codes used for the present analysis can be made available on a case-by-case basis with approval from the senior author of this manuscript. Data described in the manuscript will not be made publicly available. Further information including the procedures for obtaining and accessing data from the Nurses' Health Studies II is described online (https://www.nurseshealthstudy.org/researchers; email nhsaccess@channing.harvard.edu).
